# Diversity recovery and probiotic shift of gastric microbiota in functional dyspepsia patients after *Helicobacter pylori* eradication therapy

**DOI:** 10.3389/fmicb.2023.1288920

**Published:** 2023-11-08

**Authors:** Wenxue Wang, Zhongjian Liu, Yu Zhang, Zhiping Guo, Jieyu Liu, Siyun Li, Jihua Huang, Jiawei Geng, Fan Zhang, Qiang Guo

**Affiliations:** ^1^Department of Infectious Disease and Hepatic Disease, First People’s Hospital of Yunnan Province, Affiliated Hospital of Kunming University of Science and Technology, Kunming, Yunnan, China; ^2^School of Medicine, Kunming University of Science and Technology, Kunming, Yunnan, China; ^3^Institute of Basic and Clinical Medicine, First People’s Hospital of Yunnan Province, Affiliated Hospital of Kunming University of Science and Technology, Kunming, Yunnan, China; ^4^Department of Gastroenterology, First People’s Hospital of Yunnan Province, Affiliated Hospital of Kunming University of Science and Technology, Kunming, Yunnan, China; ^5^Department of Gastroenterology, Third People’s Hospital of Yunnan Province, Kunming, Yunnan, China; ^6^Faculty of Life Science and Technology, Kunming University of Science and Technology, Kunming, Yunnan, China

**Keywords:** functional dyspepsia, gastric microbe, microbial diversity, *Helicobacter pylori*, eradication therapy

## Abstract

The effects of *Helicobacter pylori* eradication on gastric mucosa-colonizing microbes in patients with functional dyspepsia (FD) remain unclear. Here, we explored microbial variation induced by *H. pylori* infection and eradication treatment in FD patients. Gastric microbial abundance and diversity were significantly reduced in the *H. pylori*-infected FD patients. Eradication treatment increased alpha and beta diversity of gastric mucosa-colonizing microbes, and promoted the expansion of several probiotic microbes, such as *Leuconostoc mesenteroides*, which exhibited a matched antagonistic performance against *H. pylori*. Significant variation was observed in gastric mucosa-colonizing microbes between *H. pylori*-positive and *H. pylori*-negative FD patients. Eradication treatment induced microbial diversity recovery and may provide sufficient nutrition and space for probiotic microbes, such as *Leuconostoc mesenteroides*.

## Introduction

*Helicobacter pylori* infects more than 50% of the world’s population, and its histological detection in gastric tissue has a sensitivity and specificity of over 95% (Crowe, 2019). *Helicobacter pylori* community flourishing not only induces gastric microbial imbalance, but also subsequent alimentary diseases. For example, *Helicobacter pylori* infection is an important contributor of *H. pylori* associated dyspepsia, and therefore eradication is recommended as first-line therapy in infected FD patients (Talley and Ford, 2015). The recent meta-analysis, conducted by Ayesha and colleagues, further showed that postinfectious FD patients had increased duodenal eosinophils, compared with control and *H. pylori*-negative FD patients. This study also suggested further studies were required to elevate the quality of evidences (Shah et al., 2022).

Another recent meta-analysis, conducted by Alexander and colleagues, indicates that *H. pylori* eradication therapy leads to both cure and improvement in FD symptoms (Ford et al., 2022). But complete eradication of *H. pylori* is extremely difficult, even with the use of combined and repeated medications (Chey et al., 2022; Hawkey et al., 2022; Malfertheiner et al., 2023). Correcting microbial imbalance appears to be more effective against recurrent *H. pylori* infection and overgrowth (Ford et al., 2020), although the underlying mechanism remains largely unknown. Therefore, it is crucial to study the structural characteristics of gastric microbiota in FD patients before and after eradication treatment.

As *H. pylori* infection is an important contributor of *H. pylori* associated dyspepsia, *H. pylori*-positive and *H. pylori*-negative FD patients are often prescribed different treatments (Suzuki and Moayyedi, 2013). In current clinical guidelines, sustained dyspepsia control after successful eradication identifies *H. pylori* as the organic cause, thus *H. pylori*-associated dyspepsia is considered as a separate clinical entity. Meanwhile, patients with *H. pylori* gastritis and persistent dyspepsia despite eradication therapy eliminating the infection were identified as having FD (Moayyedi et al., 2017; Miwa et al., 2022). Therefore, comparing the gastric microbiota in FD patients with and without *H. pylori* infection may provide valuable clues for overcoming recurrent *H. pylori* infection.

In addition, Moniente et al. reviewed histamine-producing bacteria (HPB), most of which colonize the human digestive tract (Moniente et al., 2021). Dual blockade of histamine receptors can be a highly effective treatment for *H. pylori*-positive FD patients, although its microbial mechanism is still unclear (Potter et al., 2020). Furthermore, while eradication drugs can induce dysbiosis of gut commensal bacteria, including HPB, the effects of *H. pylori* eradication on bacterial community structure and HPB in FD patients remain largely unknown.

This study aims to explore gastric microbial variation between *H. pylori*-negative and *H. pylori*-positive FD patients, and these *H. pylori*-positive FD patients before and after *H. pylori* eradication therapy. Our study contributes more evidences of gastric microbial shift induced by *H. pylori* infection and eradication therapy, that may profoundly influence FD progression.

## Materials and methods

### Subjects

The FD patients were recruited from the First People’s Hospital of Yunnan Province and Third People’s Hospital of Yunnan Province, China, from May 2019 to August 2020. Each FD patient received FD therapy but no use of antibiotics (within 3 months) and PPI/H2 inhibitors/NSIADs. All study protocols and procedures were approved by the Medical Ethics Board of the First People’s Hospital of Yunnan Province (GXBSC-2021001, 2021 updated), China, and were carried out in accordance with all relevant provincial, national, and international guidelines, including the Declaration of Helsinki. Written informed consent was obtained from all participants prior to their inclusion in the study.

### Diagnosis of FD and *Helicobacter pylori* eradication

We used clinical feature inquiry, laboratory examination, and imaging analysis (e.g., ultrasound or computed tomography (CT)) to diagnose FD according to clinical practice guidelines (Moayyedi et al., 2017; The Committee of Digestive System Diseases, Chinese Association of Integrative Medicine, 2017; Miwa et al., 2022). Based on ROME IV- criteria, the patients were diagnosed as FD when they showed one or more the following symptoms including postprandial fullness, early satiation, epigastric pain and epigastric burning via above tests, and had no evidence of structural disease meanwhile. The symptoms had been present for the 3 months before diagnosis and symptom onset have preceded diagnosis by at least 6 months (Stanghellini et al., 2016). Gastroscopy was performed prior to eradication, and a mucosal biopsy was taken from the gastric antrum in FD patients. The C-urea breath test was applied to identify FD patients infected with *H. pylori*, with positively infected patients given bismuth quadruple therapy consisting of rabeprazole (10 mg bid), clarithromycin (250 mg bid), amoxicillin (500 mg bid), and colloidal bismuth pectin (300 mg bid) for 14 days (Liu et al., 2018; Zhou et al., 2022). For the patients with an allergy to penicillin, omeprazole was given. Twelve months later, patients underwent gastroscopy again for mucosal collection of gastric antrum and disease progression investigation. All samples were collected from May 2019 to August 2020. Upon collection, the mucosal samples were immediately placed on ice and frozen at −80°C within 1 h for microbiome analyses.

### DNA extraction and Illumina MiSeq sequencing

Microbial community genomic DNA was extracted from gastric mucosa using a QIAamp DNA Mini Kit (Qiagen, Germany) according to the manufacturer’s instructions. The DNA extract was checked on 1% agarose gel, and DNA concentration and purity were determined with a NanoDrop 2000 UV–vis spectrophotometer (Thermo Scientific, Wilmington, USA). The hypervariable V3–V4 region of the bacterial 16S rRNA gene was amplified with primer pairs 338F (5′-ACTCCTACGGGAGGCAGCAG-3′) and 806R (5′-GGACTACHVGGGTWTCTAAT-3′) using an ABI GeneAmp® 9700 PCR thermocycler (ABI, CA, USA). Polymerase chain reaction (PCR) amplification of the 16S rRNA gene was performed in triplicate. Purified amplicons were pooled in equimolar concentrations and paired-end sequenced on the Illumina MiSeq PE300 platform (Illumina, San Diego, USA) using standard protocols.

### Processing of sequencing data

The raw 16S rRNA gene sequencing reads were demultiplexed, quality-filtered by fastp v0.20.0 (Chen et al., 2018), and merged using FLASH v1.2.7 (Magoč and Salzberg, 2011). Operational taxonomic units (OTUs) with 97% similarity cutoff (Stackebrandt and Goebel, 1994; Edgar, 2013) were clustered using UPARSE v7.1, and chimeric sequences were identified and removed. The taxonomy of each OTU representative sequence was analyzed using RDP Classifier v2.2 (Wang et al., 2007) against the 16S rRNA database (Silva v132) with a confidence threshold of 0.7. To remove possible contamination, we sequenced three samples of pure water as a negative control with the same procedures, including DNA extraction, PCR amplification, cDNA library construction, and final sequencing.

### Bioinformatics and statistical analysis

#### Alpha diversity analysis

Community richness (Chao) and diversity indices (Shannon) were used to estimate alpha diversity, which was checked by Student’s *t*-test and displayed in an estimation plot.

#### Abundance difference analysis

Significant difference of bacteria abundance uses Wilcoxon rank-sum test to apply hypothesis on genus (top 21) and phylum (top 7 or 8) levels of the microbial community of different groups of FD patients according to the community abundance data. False discovery rate (FDR) and Tukey–Kramer methods (CI = 0.95) were used for multiple testing corrections and *post-hoc* tests, respectively.

#### Phenotypic prediction of gastric mucosa-colonizing bacteria

Gastric mucosa-colonizing bacterial phenotypes were predicted and compared with BugBase (Ward et al., 2017) using the Kruskal-Wallis H test. Briefly, BugBase used the OTU table as an input file. The predicted 16S copy number was used to normalize the OTU table, with the preprocessed database and BugBase tool then used to automatically select thresholds to predict bacterial phenotypes.

#### LEfSe analysis

The nonparametric factorial Kruskal-Wallis sum-rank test was applied to detect features with significant differential abundance with respect to the class of interest. Biological significance was subsequently investigated using a set of pairwise tests among subclasses based on the Mann–Whitney-Wilcoxon test. Lastly, LEfSe analysis was applied to estimate the effect size of each differentially abundant feature and perform dimensionality reduction (Segata et al., 2011). The threshold of the logarithmic linear discriminant analysis (LDA) score for distinguishing features was set to 3.0.

#### Non-metric multidimensional scaling analysis

To reveal differences in the gastric mucosal bacterial communities in FD patients, UniFrac-weighted NMDS was performed based on the Bray–Curtis dissimilarity matrix using R v3.1.1 software.

#### Analysis of similarities

The vegan package in R was used for analysis and python was used for plotting. The R2 value obtained by ANOSIM represents the interpretation degree of sample differences between groups, i.e., ratio of group variance to total variance. Larger R2 values indicate that grouping has a higher interpretation degree to the difference, and *p* < 0.05 indicates high test reliability.

## Results

### *Helicobacter pylori* enrichment significantly decreased abundance of other gastric microbes in FD patients

**Figure 1:**
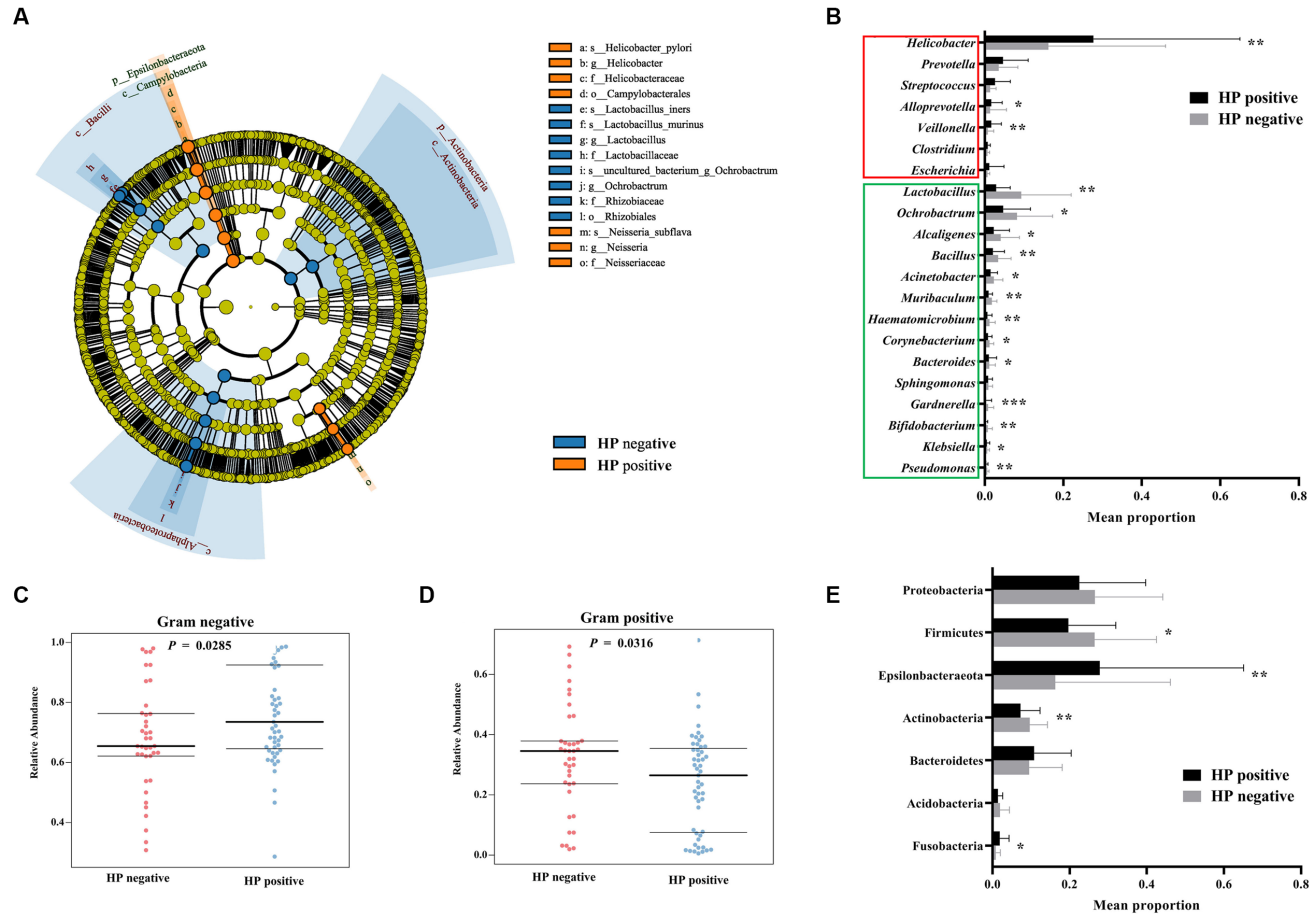
Differences in gastric bacterial abundances between *H. pylori*-positive and *H. pylori*-negative FD patients. LEfSe cladogram of potential biomarkers between *H. pylori*-positive and negative FD patients **(A)**. Wilcoxon rank-sum test comparing average composition of gastric mucosa-colonizing bacteria at genus **(B)** and phylum **(E)** level, CI = 0.95. *0.01 < *p* ≤ 0.05, **0.001 < *p* ≤ 0.01, ***p ≤ 0.001. BugBase predicted gram negative **(C)** and positive **(D)** phenotypes of gastric mucosa-colonizing bacteria, CI = 0.95. *H. pylori* negative: n = 45; *H. pylori* positive: *n* = 54.

Of the 98 recruited FD patients, 54 (55%) were *H. pylori*-positive based on the C-13 urea breath test and the numbers needed to treat (NNT) with eradication therapy was 44 (95% CI 39 to 62). The basic characteristics, occupations, and chief complaints of these patients are shown in Table 1. *LEfSe* analysis revealed that gram-negative proteobacterium *Neisseria subflava* was a taxonomic biomarker of *H. pylori*-positive FD patients. Analysis also indicated that *Lactobacillus* (including *Lactobacillus murinus* and *Lactobacillus iners*) and *Ochrobactrum* were taxonomic biomarkers of *H. pylori*-negative FD patients (Figure 1A). BugBase phenotypic analysis showed that *H. pylori*-positive FD patients had decreased total gram-positive bacterial abundance and increased total gram-negative bacterial abundance compared to *H. pylori*-negative patients (Figures 1C,D).

**Table 1:** Basic characteristics of functional dyspepsia patients.

Index	*H. pylori* positive (*n* = 44)	*H. pylori* negative (*n* = 44)
Gender (male: female)	25:19	17:27
Age (mean ± SD)	48.74 ± 12.28	46.14 ± 12.46
Smoking (yes: no)	15:29	10:34
Antibiotic use (within 1 month)	No	No
Occupation		
Farmer	11	13
Factory worker	25	21
Office staff	2	8
Others	6	2
Chief complaint		
Epigastric pain	19	21
Epigastric burning	11	13
Postprandial fullness	5	1
Early satiety	4	2
Others	5	7

In addition to *H. pylori* and *Neisseria subflava*, other gram-negative bacteria also showed increased abundances in the *H. pylori*-positive FD patients, including *Escherichia* and *Veillonella*. Gram-positive bacteria, such as *Lactobacillus*, *Bacillus*, *Corynebacterium*, and *Bifidobacterium*, showed decreased abundances in the *H. pylori*-positive FD patients (Figure 1B). Of note, most of the gram-positive bacteria (*Lactobacillus*, *Bacillus*, and *Bifidobacterium*) exhibit probiotic potential, and *H. pylori* overgrowth may deprive these bacteria of essential nutrients and space.

Most bacterial phyla (e.g., Proteobacteria, Firmicutes, Actinobacteria, and Acidobacteria) showed decreased abundances in the *H. pylori*-positive FD patients, except for Fusobacterium and Epsilonbacteraeota (Figures 1A,E).

### *Helicobacter pylori* eradication increased gastric microbial alpha and beta diversity in FD patients

**Figure 2:**
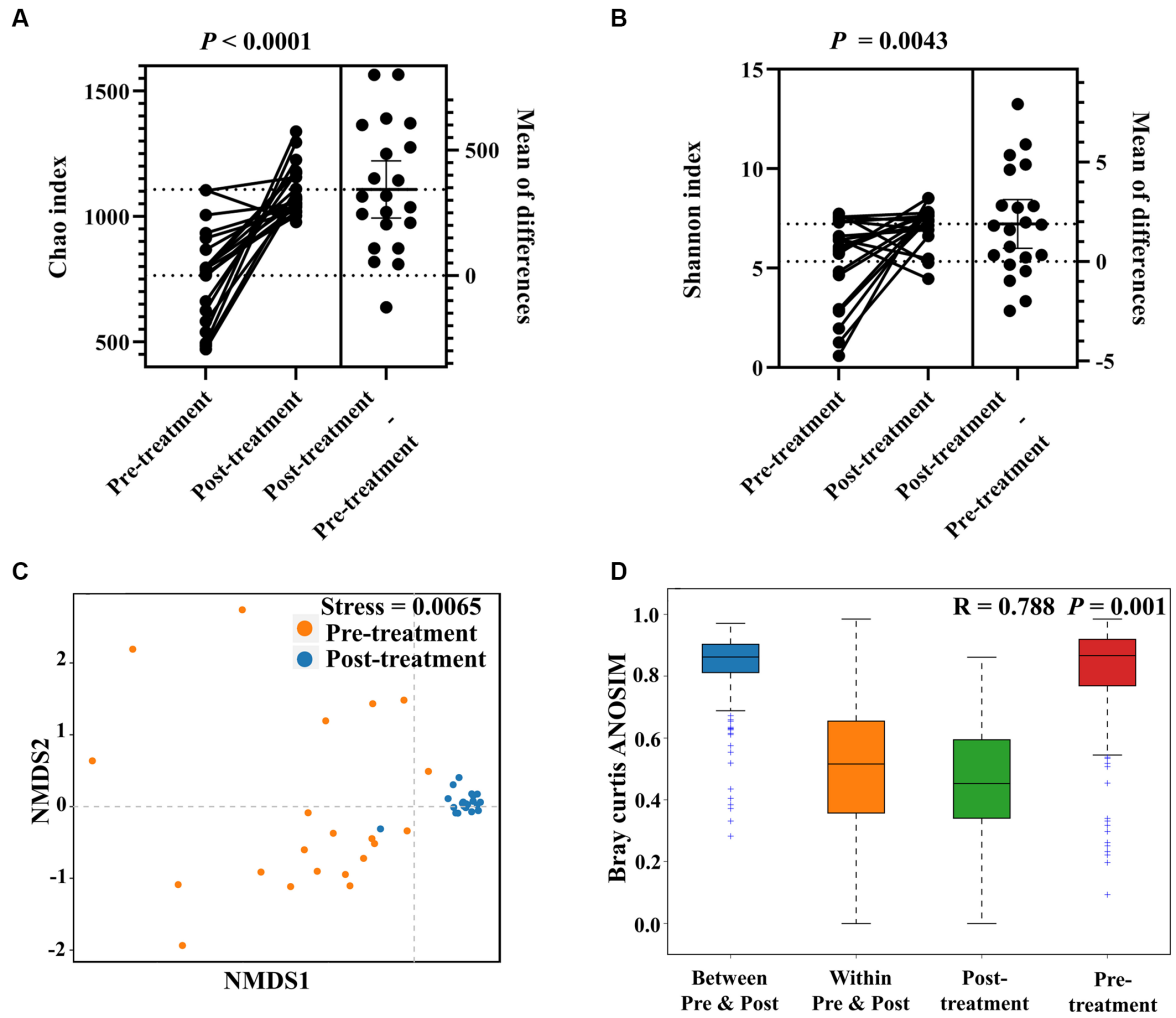
Effects of *H. pylori* eradication on gastric microbial diversity in FD patients. Alpha diversity of gastric mucosa-colonizing microbiota based on Chao index **(A)** and Shannon index **(B)**. Unweighted UniFrac NMDS of gastric mucosa-colonizing microbiota **(C)**. ANOSIM box plot based on unweighted UniFrac distance showing significant differences between pre-treatment (before *H. pylori* eradication) and post-treatment (after HP eradication) groups **(D)**.

The *H. pylori*-positive FD patients were treated with bismuth quadruple therapy. Twelve months after treatment, gastric mucosa were collected by endoscopy for microbiota diversity analysis. After eradication therapy, these patients were identified to be *H. pylori*-negative via C-urea breath test, and patient’s complaints were notably relieved (epigastric pain: 68% (13/19); epigastric burning: 64% (7/11); postprandial fullness: 80% (4/5); early satiety: 50% (2/4)). Alpha diversity analysis indicated that *H. pylori* eradication significantly increased the Chao (765.40 ± 188.10 to 1108.10 ± 95.55, *p* < 0.0001) and Shannon indices (5.34 ± 2.17 to 7.23 ± 1.03, *p* = 0.0043) of gastric mucosa-colonizing bacteria in the FD patients (Figures 2A,B). NMDS analysis showed that *H. pylori* eradication significantly homogenized the structure of the gastric mucosa-colonizing microbiota in the FD patients (Stress = 0.0065; Figure 2C). ANOSIM further confirmed that *H. pylori* eradication markedly transformed the structure of the gastric mucosa-colonizing microbiota in the FD patients (R = 0.823, *p* = 0.001; Figure 2D).

### *Helicobacter pylori* eradication differentiated species and their potential functions

**Figure 3:**
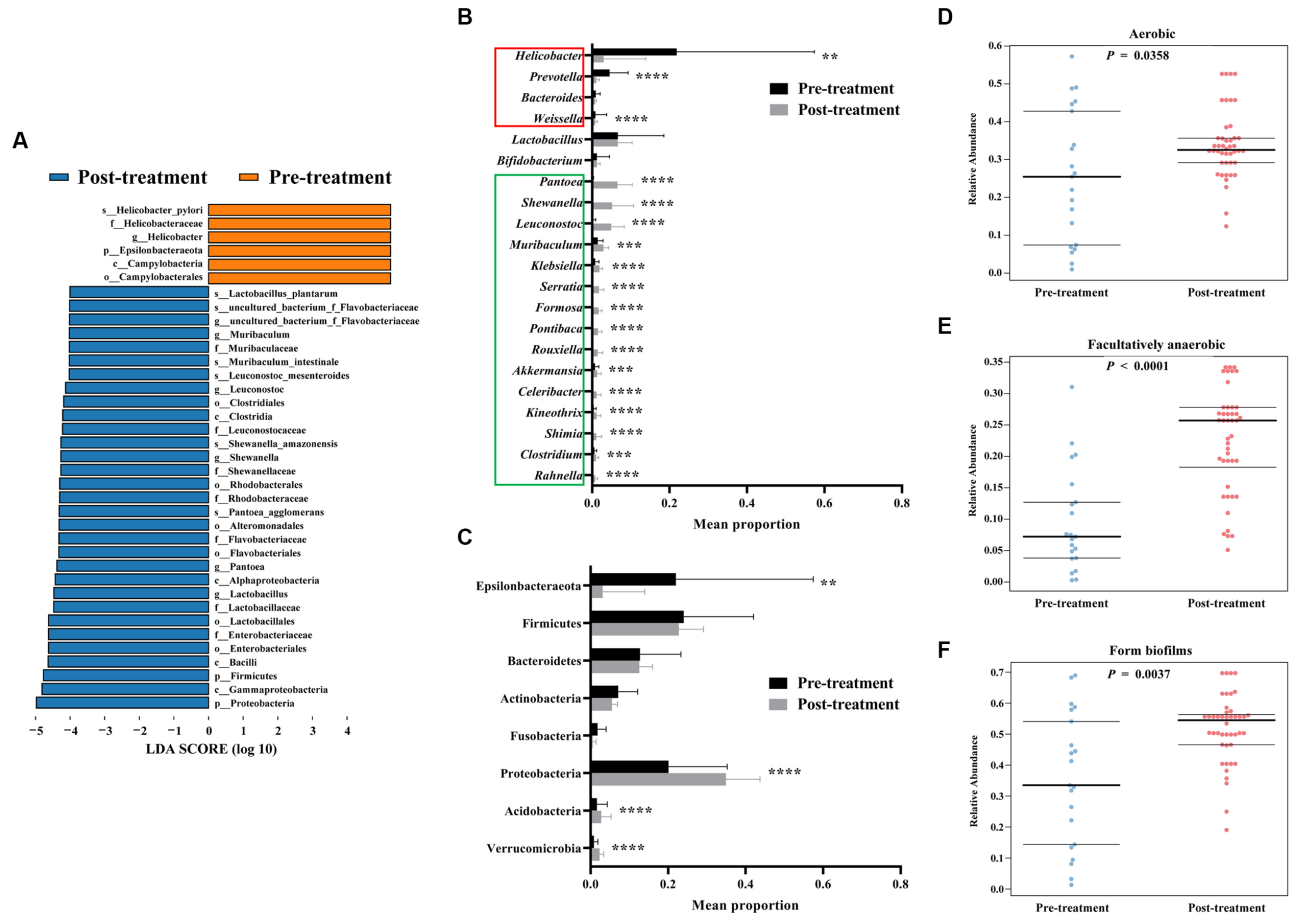
Increased gastric bacterial abundance after *H. pylori* eradication in FD patients. LEfSe histogram of LDA scores for differentially abundant features between pre-treatment (before HP eradication) and post-treatment (after *H. pylori* eradication) FD patients **(A)**. Threshold of logarithmic LDA score for discriminative features was set to 4.0. Wilcoxon rank-sum test comparing average composition of gastric mucosa-colonizing bacteria at genus **(B)** and phylum **(C)** level, CI = 0.95. *0.01 < *p* ≤ 0.05, **0.001 < *p* ≤ 0.01, ***p ≤ 0.001, ****p ≤ 0.0001. BugBase predicted aerobic **(D)**, facultatively anaerobic **(E)**, and biofilm-forming **(F)** phenotypes of gastric mucosa-colonizing bacteria, CI = 0.95.

In the *H. pylori*-positive FD patients, only *H. pylori* exihibited a significant LDA score. After *H. pylori* eradication by bismuth quadruple therapy, the taxonomic biomarkers changed to *Lactobacillus* (*Lactobacillus plantarum*), *Leuconostoc* (*Leuconostoc mesenteroides*), *Muribaculum*, *Shewanella* (*Shewanella amazonensis*), and *Pantoea* (*Pantoea agglomerans*), indicating complete alteration in the structure of the gastric microbiota after *H. pylori* eradication (Figure 3A).

Specifically, *H. pylori* eradication significantly decreased the abundances of *Weissella* and *Prevotella*, which all possess pathogenic potential. Eradication significantly increased multiple bacteria, including *Pantoea*, *Shewanella*, *Leuconostoc*, *Muribaculum*, *Klebsiella*, *Serratia*, *Formosa*, *Pontibaca*, *Rouxiella*, *Akkermansia*, *Celeribacter*, *Kineothrix*, *Shimia*, *Clostridium*, and *Rahnella*. Fortunately, the abundances of bacteria known to belong to probiotic genera *Bifidobacterium* and *Lactobacillus* were not significantly changed by *H. pylori* eradication (Figure 3B). At the phylum level, Proteobacteria, Acidobacteria, and Verrucomicrobia showed increased abundances (Figure 3C).

Furthermore, *H. pylori* eradication appears to increase the abundances of non-anaerobic bacteria. Functional prediction analysis indicated that the abundances of both aerobic and facultatively anaerobic bacteria increased significantly (aerobic: 0.25 ± 0.17 to 0.34 ± 0.09, *p* = 0.0358; aerobic: 0.09 ± 0.08 to 0.23 ± 0.26, *p* < 0.0001) after eradication treatment (Figures 3D,E). However, treatment also increased the abundances of biofilm-forming bacteria (0.35 ± 0.33 to 0.52 ± 0.54, *p* = 0.0037), which may impact biofilm-based antibiotic resistance and tolerance (Figure 3F).

### Gastric bacterium of FD patients after *Helicobacter pylori* eradication can be divided into two subgroups in *Leuconostoc mesenteroides*-dependent manner

**Figure 4:**
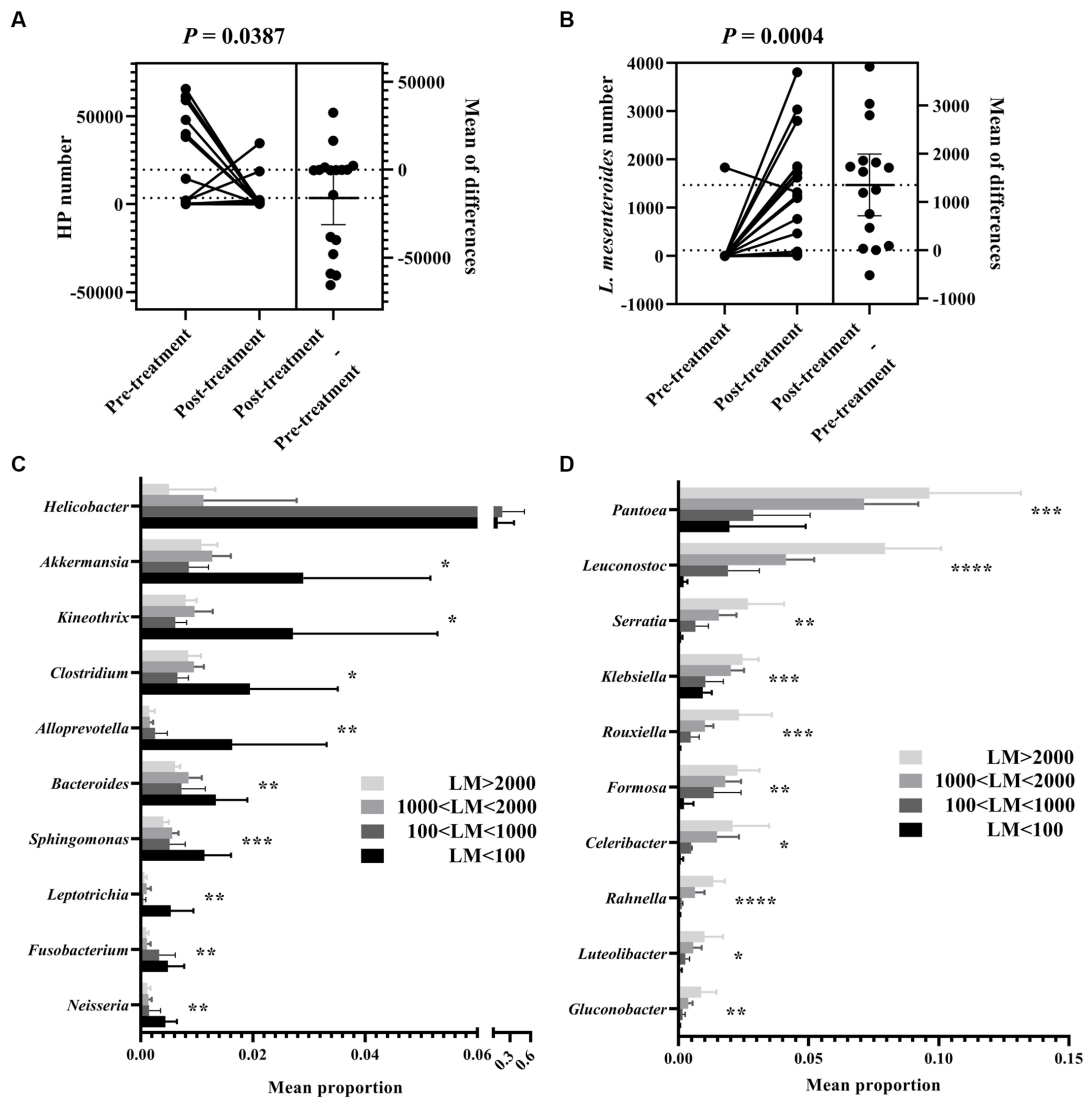
*Leuconostoc mesenteroides* abundance-dependent grouping of gastric bacteria in FD patients after *H. pylori* eradication. Abundance comparison of *H. pylori* **(A)** and *L. mesenteroides* **(B)** between pre-treated and post-treated FD patients. Wilcoxon rank-sum test comparing average composition of gastric mucosa-colonizing bacteria, divided into two subgroups, i.e., *H. pylori* l dominant and **(C)** and *L. mesenteroides* dominant **(D)** in FD patients after *H. pylori* eradication. LM < 100, *L. mesenteroides* number less than 100; 100 < LM < 1,000, *L. mesenteroides* number greater than 100, but less than 1,000; 1,000 < LM < 2,000, *L. mesenteroides* number greater than 1,000, but less than 2,000; LM > 2000, *L. mesenteroides* number lower than 2,000. CI = 0.95. *0.01 < *p* ≤ 0.05, **0.001 < *p* ≤ 0.01, ***p ≤ 0.001, ****p ≤ 0.0001.

We further explored changes in gastric bacterial composition induced by eradication treatment. Results showed that *H. pylori* abundance was markedly decreased (Figure 4A), while *Leuconostoc mesenteroides* abundance was markedly increased (Figure 4B) after eradication treatment. This change in abundance of core bacteria indicated a fundamental shift in the gastric microbiota. Therefore, we divided the FD patients into two subgroups according to *Leuconostoc mesenteroides* abundance after eradication. Interestingly, nine core genera (i.e., *Pantoea*, *Serratia*, *Klebsiella*, *Rouxiell*a, *Formosa*, *Celeribacter*, *Rahnella*, *Luteolibacter*, and *Gluconobacter*) showed a significant increase in abundance with the increase in *Leuconostoc mesenteroides* (Figure 4D), while 10 core genera (i.e., *Helicobacter*, *Akkermansia*, *Kineothrix*, *Clostridium*, *Alloprevotella*, *Bacteroides*, *Sphingomonas*, *Leptotrichia*, *Fusobacterium*, and *Neisseria*) showed a significant decreased in abundance (Figure 4C).

## Discussion

Modulation of the microbiome in the stomach or small intestine is an important strategy for the management of *H. pylori*-positive FD patients (Ford et al., 2020). In the current study, *H. pylori*-positive FD patients showed increased levels of gram-negative bacteria, whereas *H. pylori*-negative FD patients showed increased levels of gram-positive bacteria, including probiotics such as *Lactobacillus* and *Bifidobacterium*. *Helicobacter pylori*-induced overgrowth of gram-negative bacteria may reduce space for probiotics, indirectly leading to dyspeptic symptoms. However, the initial drivers of gram-negative bacterial overgrowth remain to be explored.

Although *H. pylori* eradication has clear benefits in regard to improving FD symptoms (Ford et al., 2022), microbial diversity variation after treatment remains controversial. In our study, the Chao and Shannon indices of gastric mucosa-colonizing microorganisms were significantly increased in FD patients 12 months after eradication therapy. Most chief complaints of these patients were significantly released, including epigastric pain, epigastric burning and postprandial fullness. Companying these phenotypes, *H. pylori*’s dominance was replaced by *Lactobacillus* and *Leuconostoc*. Even there is no evidences of this dominance-replacing is a direct contributor of complaint relief, we recommend they are connected directly or indirectly.

Moreover, microbes that colonize different vertical layers of the gastric mucosa may be affected differently by therapeutic drugs due to distinct drug exposure. When drug exposure eradicates most non-drug resistant microbes, *H. pylori* may obtain superior nutrition and space. Phenotype prediction analysis showed increased abundance of aerobic microbes (including facultatively anaerobic) after eradication treatment. However, it is unclear whether this was due to variation in oxygen concentration of different vertical layers of the gastric mucosa or eradication by drug-specific targeting. On the other hand, the limited effects of eradication treatment may originate from incomplete deletion of *H. pylori* that colonize different vertical layers of the gastric mucosa, partially at least.

Histamine contributes to FD progression (Moniente et al., 2021), and blockade of its receptors induces a marked improvement in FD patients (Potter et al., 2020). Previous research has shown that *Leuconostoc mesenteroides* can scavenge 40% of amine-containing compounds by producing exopolysaccharides (Li et al., 2020). *Leuconostoc mesenteroides* can also effectively degrade histamine by over 85% (Lee et al., 2021). Our study showed that *H. pylori* eradication led to a significant increase in *Leuconostoc mesenteroides*, which exhibited an obvious antagonistic effect on microbial abundance of *H. pylori*. Thus, the dominant microbes were basically divided into two subgroups, and their abundances were closely correlated with either *H. pylori* or *Leuconostoc mesenteroides*. These findings would be more convincing if there was no limitation on the absence of healthy population as a control group. However, our data still remind physicians pay attention to *Leuconostoc mesenteroides* during FD patient recovery progress.

Meanwhile, our study has several limitations. Most FD patients were factory workers (*n* = 46) and farmers (*n* = 24) and came from same area, Kunming, China (*n* = 83). Their similar living and working environments may affect gastric microbiota components, leading to a microecosystem bias. The further study also should clarify the relationship between the characteristics of changes in gastric microbiota before and after *H. pylori* eradication and changes in FD symptoms.

In conclusion, our study revealed significant variation in gastric mucosa-colonizing microbes between *H. pylori*-positive and *H. pylori*-negative FD patients. Eradication treatment resulted in restoration of microbial diversity and improvement in dyspeptic symptoms, *Leuconostoc mesenteroides* may contribute to this improvement.

## Data availability statement

The datasets presented in this study can be found in online repositories. The names of the repository/repositories and accession number(s) can be found here: https://www.ncbi.nlm.nih.gov/, PRJNA843681.

## Ethics statement

The studies involving humans were approved by the Medical Ethics Board of the First People’s Hospital of Yunnan Province. The studies were conducted in accordance with the local legislation and institutional requirements. The participants provided their written informed consent to participate in this study.

## Author contributions

WW: Conceptualization, Funding acquisition, Investigation, Supervision, Validation, Writing – review & editing, Formal Analysis, Project administration, Software, Writing – original draft. ZL: Conceptualization, Formal Analysis, Funding acquisition, Software, Validation, Writing – review & editing. YZ: Validation, Writing – review & editing, Investigation, Methodology, Visualization. ZG: Investigation, Methodology, validation, Writing – review & editing, Resources. JL: Investigation, Methodology, Validation, Writing – review & editing, Project administration, Visualization. SL: Investigation, Methodology, Validation, Visualization, Writing – review & editing. JH: Investigation, Methodology, Validation, Visualization, Writing – review & editing. JG: Investigation, Validation, Writing – review & editing, Conceptualization, Funding acquisition, Supervision. FZ: Funding acquisition, Conceptualization, Investigation, Methodology, Project administration, Resources, Supervision, Validation, Visualization, Writing – review & editing, Data curation. QG: Writing – original draft, Conceptualization, Investigation, Methodology, Project administration, Resources, Supervision, Validation, Visualization, Writing – review & editing.
