# Corrigendum: Diversity recovery and probiotic shift of gastric microbiota in functional dyspepsia patients after *Helicobacter pylori* eradication therapy

**DOI:** 10.3389/fmicb.2023.1354558

**Published:** 2024-01-08

**Authors:** Wenxue Wang, Zhongjian Liu, Yu Zhang, Zhiping Guo, Jieyu Liu, Siyun Li, Jihua Huang, Jiawei Geng, Fan Zhang, Qiang Guo

**Affiliations:** ^1^Department of Infectious Disease and Hepatic Disease, First People's Hospital of Yunnan Province, Affiliated Hospital of Kunming University of Science and Technology, Kunming, Yunnan, China; ^2^School of Medicine, Kunming University of Science and Technology, Kunming, Yunnan, China; ^3^Institute of Basic and Clinical Medicine, First People's Hospital of Yunnan Province, Affiliated Hospital of Kunming University of Science and Technology, Kunming, Yunnan, China; ^4^Department of Gastroenterology, First People's Hospital of Yunnan Province, Affiliated Hospital of Kunming University of Science and Technology, Kunming, Yunnan, China; ^5^Department of Gastroenterology, Third People's Hospital of Yunnan Province, Kunming, Yunnan, China; ^6^Faculty of Life Science and Technology, Kunming University of Science and Technology, Kunming, Yunnan, China

**Keywords:** functional dyspepsia, gastric microbe, microbial diversity, *Helicobacter pylori*, eradication therapy

In the published article, an author name was incorrectly written as Fang Zhang. The correct spelling is Fan Zhang.

The authors apologize for this error and state that this does not change the scientific conclusions of the article in any way. The original article has been updated.

